# Survival strategy of *Pseudomonas aeruginosa* on the nanopillar topography of dragonfly (*Pantala flavescens*) wing

**DOI:** 10.1186/s13568-020-01021-7

**Published:** 2020-05-06

**Authors:** Banu Pradheepa Kamarajan, Ananthasubramanian Muthusamy

**Affiliations:** Department of Biotechnology, PSG College of Technology, Coimbatore, TamilNadu India

**Keywords:** *Pseudomonas aeruginosa*, Dragonfly wing, Nanopillar surface, Adhesion

## Abstract

Discovery of nanopillars on the surface of the insect wings had led to the understanding of its bactericidal property. Nanopillar topography is deterrent to only those bacteria that are attached, or in close contact with the nanopillars. The present study investigated the variation in the viability of *Pseudomonas aeruginosa* strains PAO1 (virulent) and ATCC 9027 (avirulent) on the wing surface of dragonfly (*Pantala flavescens*). Viability study indicated that only 0.2% ATCC 9027 survived when incubated with wing for 48 h in Phosphate buffered saline, while under the same conditions 43.47% PAO1 survived. Enumeration of Pseudomonas attached to wing surface suggested that, the number of PAO1 attached on the wing surface was three times lesser than ATCC 9027. Propensity of attachment of *P. aeruginosa* strains PAO1 and ATCC 9027 on the wing surface investigated using scanning probe microscope indicated that *P. aeruginosa* ATCC 9027 showed adhesion to 88% of regions and, PAO1 showed adhesion to only 48% regions tested on wing surface. PAO1 survived the bactericidal effect of wing surface by evading attachment. Three clinical isolates tested which showed viability similar to PAO1 strain, also showed lower propensity to attach to wing surface. Transcriptional level analyses using RT-PCR suggested that flagellar genes (fliE and fleS) were downregulated and genes responsible for reversible to irreversible attachment (gcbA and rsmZ) were upregulated in ATCC 9027 than PAO1 on wing surface, indicating relatively higher attachment of ATCC 9027 on wing surface. The study suggests that virulent strains of *P. aeruginosa* may evade attachment on wing surface. The results gain significance as bioinspired surfaces are being created towards developing antibacterial medical implants and other antibacterial surface applications.

## Key points

1. Survival of PAO1 was nearly 45% on the wings of dragonfly.
2. PAO1 has lower propensity for attachment on dragonfly wing surface.
3. Lesser the attachment of *P. aeruginosa* on wing surface, higher is the survival.

## Introduction

Bacterial colonization on the medical implants could be life-threatening. To prevent bacterial colonization on medical implants, conventional techniques such as coating the implant material with antibiotics (Romanò et al. 2015), coating with functional DNase I (Swartjes et al. 2013), coating with glycoside hydrolase (Asker et al. 2018) controlled release of antibiotics from the implant material (Prabu and Kim 2008) and, use of contact-killing implant surfaces (Tiller et al. 2001) are being followed. The pitfalls of implant coating are limited shelf-life, cold-chain requirement and, drug resistance. To combat this situation, strategies that use physical means to deter bacterial colonization rather than use of antibiotics would be desirable, as the chances of bacteria developing resistance against the physical means is most unlikely.

Bioinspired structures are being studied as an alternative approach to restrict bacterial colonization. Nanopillars on the wings of cicada (Ivanova et al. 2012), dragonfly (Ivanova et al. 2013), damselfly (Truong et al. 2017), larvae of drone fly (Hayes et al. 2016), sanddragon dragon fly and feet of gecko lizard (Tripathy et al. 2017) were identified to possess bactericidal property against numerous Gram negative bacteria such as *E. coli, P. aeruginosa, P. fluorescens, K. pneumoniae, P. ginigivalis, Salmonella, Camphylobacter* and, Gram positive bacteria such as *S. aureus, B. subtilis, E. fecalis, Listeria,* and *C. perfringens* (Tripathy et al. 2017) and spores of *Bacillus subtilis* (Ivanova et al. 2013). Bactericidal property was also observed on nanotopography mimicked on the artificial surfaces such as black silicon (Ivanova et al. 2013), titanium dioxide surface (Diu et al. 2014; Bhadra et al. 2015), diamond nano cones (Fisher et al. 2016), Zinc oxide (Yi et al. 2018) and Polymethylmethaacrylate (Dickson et al. 2015), towards developing bioinspired surfaces on medical implants and other antibacterial surfaces. Numerous bactericidal surfaces were patented and were successfully translated and currently in use in orthopedic implants (Orapiriyakul et al. 2018), dental implants (Asensio et al. 2019) and nanopillar surface on artificial cornea had entered pre-clinical trials (Chough et al. 2018). Further research would facilitate development of better surfaces, with translation already into practice.

Few hypotheses were proposed for the bactericidal activity of insect wing. (1) Bactericidal activity of nanopillars was reported to be direct mechanical puncturing of the bacterial cell wall by nanopillars regardless of the material’s chemical properties (Ivanova et al. 2013). (2) Pogodin et al. (2013) via modeling proposed that *P. aeruginosa* cells stretch on the nanopillars which caused decrease in the thickness of cell wall, hence punctured by the nanopillars. (3) Bandara et al. (2017) via advanced microscopic techniques proposed that, bacteria attached to the nanopillars of dragonfly wing, when attempt to move, the cell membrane gets damaged causing the cellular contents to leak. These hypotheses suggest that the nanopillars do not discourage bacterial attachment, instead are cidal to bacteria that are attached to it. The above hypotheses also hint that, for the nanopillars topography to be bactericidal, bacteria primarily need to attach to such surface.

*Pseudomonas aeruginosa* is the bacterium most used to study the bactericidal effect of nanopillars topography. *P. aeruginosa* is marked to be critical by World Health Organization among the lethal infection causing bacteria in 2018 (Release 2018). Strains of *P. aeruginosa* such as ATCC 9027 (Ivanova et al. 2012, 2013; Hasan et al. 2013; Bhadra et al. 2015), ATCC 9721 (Truong et al. 2017) and ATCC 27853 (Diu et al. 2014; Tripathy et al. 2017) were reported to be killed on the nanopillar topography of natural and bioinspired surfaces. *P. aeruginosa* ATCC 9721 and ATCC 27853 are quality control strains as per ATCC catalogue, while ATCC 9027 is avirulent (Jayal et al. 2017; Grosso-Becerra et al. 2016). Behavior of the virulent strains on the nanopillars topography is not clearly defined. In this study, we chose PAO1, virulent strain (Attila et al. 2008) and ATCC 9027, an avirulent strain (Jayal et al. 2017; Grosso-Becerra et al. 2016) to investigate the viability, attachment and adhesion force on the nanopillar topography of the wing surface.

## Materials and methods

### Wing characterization

Dragonflies (*Pantala flavescens*) were collected from Botanical gardens in Coimbatore, India. The wings of dragonflies were rinsed with milliQ water (18.2 MΩ cm resistivity), dried in the laminar hood and stored for experiments. The wings were sputter coated using Emitech SC7620 Mini Sputter Coater and imaged using FEI Quanta 250 Field Emission Scanning electron microscope (FESEM). Water contact angle of the wing was measured using KRUSS-Drop Shape Analyzer DSA 25E. Chemical characterization was performed using Energy Dispersive X-ray spectroscopy (Bruker) and Fourier Transformed Infrared Radiation (Perkin Elmer FTIR model C96836).

### Bacterial culture preparation

*Pseudomonas aeruginosa* strain ATCC 9027 (MTCC 1688) was procured from Microbial Type Culture Collection, Chandigargh, India and, *P. aeruginosa* PAO1 was procured from National Collection of Industrial Microorganisms, National Chemical Laboratory, Pune, India. *P. aeruginosa* clinical isolates (named 1570, 1589 and 1595) were obtained from PSG Institute of Medical Sciences & Research, India.

Single colony of *P. aeruginosa* PAO1 and ATCC 9027 were separately inoculated in 5 mL of nutrient broth and cultured overnight at 37 °C shaking at 120 rpm. The cultures were centrifuged, and the pellets were resuspended in Phosphate Buffered Saline (PBS) pH 7.4 to adjust OD_600nm_ to 1.0. The above inoculums were diluted ten times with PBS. PBS does not support bacterial growth and hence control over the cell count could be achieved. Coverslip and wing were cut to 10 mm diameter. Glass coverslip was used as control as reported earlier (Ivanova et al. 2013). In 48-well plate, 1 mL culture was added to each well containing coverslip/wing and incubated for discrete time periods. The above procedure was followed for all the tests unless otherwise mentioned.

### Bacterial viability

#### Quantitative analysis of bacterial viability

Bacterial viability was analyzed using flow cytometer (BD FACS verse). 1 mL of *P. aeruginosa* cultures were incubated separately with coverslip/wing for 30 min, 1 h, 2 h, 7 h, 24 h and 48 h under static condition in 48-well plate. After incubation time, 1 mL of the cell suspensions were transferred to fresh 2 mL centrifuge tubes. Coverslip and wing were sonicated (Lab companion ultra Sonic cleaner UCP-02) to detach the cells from coverslip/wing in 0.5 mL PBS. Post-sonication, coverslip/wing was discarded and the contents were pooled to their respective 1 mL cell suspensions that were collected earlier. Cells attached to the coverslip/wing were pooled to the suspension to get cell concentration of 10^7^, which is the minimal cell count required for analyses using flow cytometry. To this pooled suspension, 10 µM propidium iodide (MP Biomedicals 195458) was added, mixed and incubated in dark for 10 min. Propidium iodide stains the dead cells red. The viable cells remain unstained. The suspensions were loaded onto the BD Facs Verse flow cytometer and medium speed was selected to analyze the cells. 10,000 events were studied. The results were obtained using BD FACSuite software application. The flow cytometry data obtained are presented in percentage and not in log scale, as it likely eliminates the low-intensity data due to signal compensation (Herzenberg et al. 2006).

#### Qualitative analysis of bacterial viability

*Pseudomonas aeruginosa* strains were incubated with coverslip/wing in 48-well plate for 24 h at 37 °C. After incubation, coverslip/wing were rinsed gently and transferred to fresh 1.5 mL centrifuge tube containing 1 mL 0.85% saline (PBS affects the efficiency of fluorescent staining, hence saline was used). Coverslip/wing in saline was sonicated to detach the cells. After sonication, coverslip/wing was discarded. The cells were stained with BacLight Live/dead bacterial fluorescent staining kit (Invitrogen L7012) as per manufacturer’s instructions. Nucleic acid of live cells were stained green using SYTO9 and dead cells red using propidium iodide (Dickson et al. 2015). 5 µL of the suspension was transferred to glass slide and imaged under fluorescent microscope (Nikon Ti eclipse, 100× oil immersion). Images were captured using NIS-Elements BR version 4.50.00 version.

#### Quantification of DNA in suspension

It is presumed that cells whose cell walls were damaged on the nanopillars release the cellular DNA into the suspension. The DNA released into the cell suspension was quantified without disturbing the cells with intact cell walls. After incubating the *P. aeruginosa* with coverslip/wing for discrete time periods, 1 mL of cell suspension was collected in fresh 2 mL centrifuge tubes. Equal volume of phenol: chloroform: isoamyl alcohol (25:24:1) were added, mixed well and incubated for 10 min at room temperature. The contents were centrifuged at 12,000×*g* for 15 min at 4 °C. The top aqueous layer was transferred to a fresh 2 mL centrifuge tube. 2.5 volume of ice cold isopropanol was added to precipitate the DNA. The contents were mixed and allowed to stand for 10 min in ice. The tubes were centrifuged at 12,000×*g* for 15 min at 4 °C to pellet the DNA. Supernatant was discarded and the DNA pellet was washed with 75% ethanol and centrifuged again. The DNA pellet was suspended in 1× Tris EDTA buffer. Using 1× TE buffer as blank, the concentrations of the DNA obtained were read using nanospectrophotometer (Quickdrop Spectramax–Micro volume spectrophotometer).

### Bacterial attachment

*Pseudomonas aeruginosa* strains were separately incubated with coverslip/wing for discrete time periods in 48-well plate. After incubation time, the coverslip and wing were rinsed gently and sonicated in 1 mL PBS. The coverslip/wing was discarded post-sonication. The cells were stained and enumerated under light microscope using hemocytometer. Triplicates were used.

### Scanning electron microscopic imaging

Bacterial attachment on the coverslip/wing was studied using scanning electron microscopy. *P. aeruginosa* strains were incubated with coverslip/wing for 2 h, 7 h and 24 h. After incubation, the cell suspension was discarded and coverslip/wing was rinsed with PBS gently. The cells on the coverslip/wing were fixed with 2.5% gluteraldehyde for 15 min. The gluteraldehyde was discarded and coverslip/wing was rinsed with PBS thrice. The samples were dehydrated gradually with increasing percentages of ethanol 30%, 50%, 70%, 90%, 100% and 100% replacing each after every 10 min (Tang et al. 2014). The samples were then gold coated using Emitech SC7620 Mini Sputter Coater and imaged in FEI Quanta 250 Scanning electron microscope.

### Adhesion force measurement

Adhesion force measurements were studied using Scanning Probe Microscope (NT-MDT). Overnight grown *P. aeruginosa* were centrifuged and the pellet was resuspended in PBS and the OD_600nm_ was adjusted to 1.0. 1 µL of the culture suspended in PBS was added to the cantilever (CSG10 series, NT-MDT) and allowed to dry at room temperature. Bacterial attachment to cantilever is carried out according to the protocol specified by Touhami et al. (2006). The cantilever with bacteria attached was mounted onto the scanning probe microscope. Meanwhile, glass coverslip and wing were cut into 1 × 1 cm^2^ and fixed onto two separate stubs. Using contact mode, the adhesion forces were measured at 25 random points on the coverslip/wing. The cantilever with the bacteria was allowed to rest on coverslip/wing for 1 s, and retracted. For each point, force-distance curves were constructed using Origin 8.5 software. The difference between the cantilever approach and retract value is the height (h). The ‘h’ value denotes the adhesion. The spring constant of the cantilever used was 0.11 N/m.

The ‘h’ values obtained were substituted in the Hooke’s law to get the adhesion force.

By Hooke’s law,

$${\text{Adhesion}}\;{\text{force}},{\text{ F}}\, = \,{\text{k }} \times \, \Delta {\text{h}}$$

where, k, the spring constant is 0.11 N/m.

Number of points to which the *P. aeruginosa* strain shows adhesion was studied. In addition, the adhesion forces exerted on each point were also calculated. The adhesion forces were plotted as a Box–Whisker graph using Origin 8.5 software.

### Biofilm forming assay

The biofilm forming ability of the *P. aeruginosa* strains were tested using the standard crystal violet assay in 96-well plate (O’Toole 2011). The absorbance values are proportional to the biofilm formed. Depending upon the absorbance values, Hassan et al. (2011) reported that bacteria can be classified as non-biofilm former (OD of test ≤ 2× OD of control), weak-moderate biofilm former (OD of test > 2× to ≤ 4× OD of control) and, potent biofilm former (OD of test > 4× OD of control).

### Real-time PCR

The PAO1 and ATCC 9027 cultures were diluted to OD_600_ of 0.1 using PBS. The cultures were incubated with wings and coverslip separately in 48-well plate at 37 °C under static condition. After 30 min, 1 h, 2 h, 7 h, 24 h and 48 h, total RNA was extracted from the cells using Tri Reagent (Sigma Aldrich Catalogue No. T9424). The total RNA was used to synthesize cDNA using ThermoFisher kit (K1622). The synthesized ss cDNA was used in Bio-rad tubes (TCS 0801, TCS 0803) with Bio-rad SYBR mix (170-8880AP) for real-time PCR as per the manufacturer’s instructions in the CFX96 Bio-rad RT-PCR instrument. The primers of the genes fliE, fleS, pelA, gcbA, rsmZ and mreB were synthesized commercially using the sequences provided by Petrova et al. (2014).

## Results

**Fig. 1:**
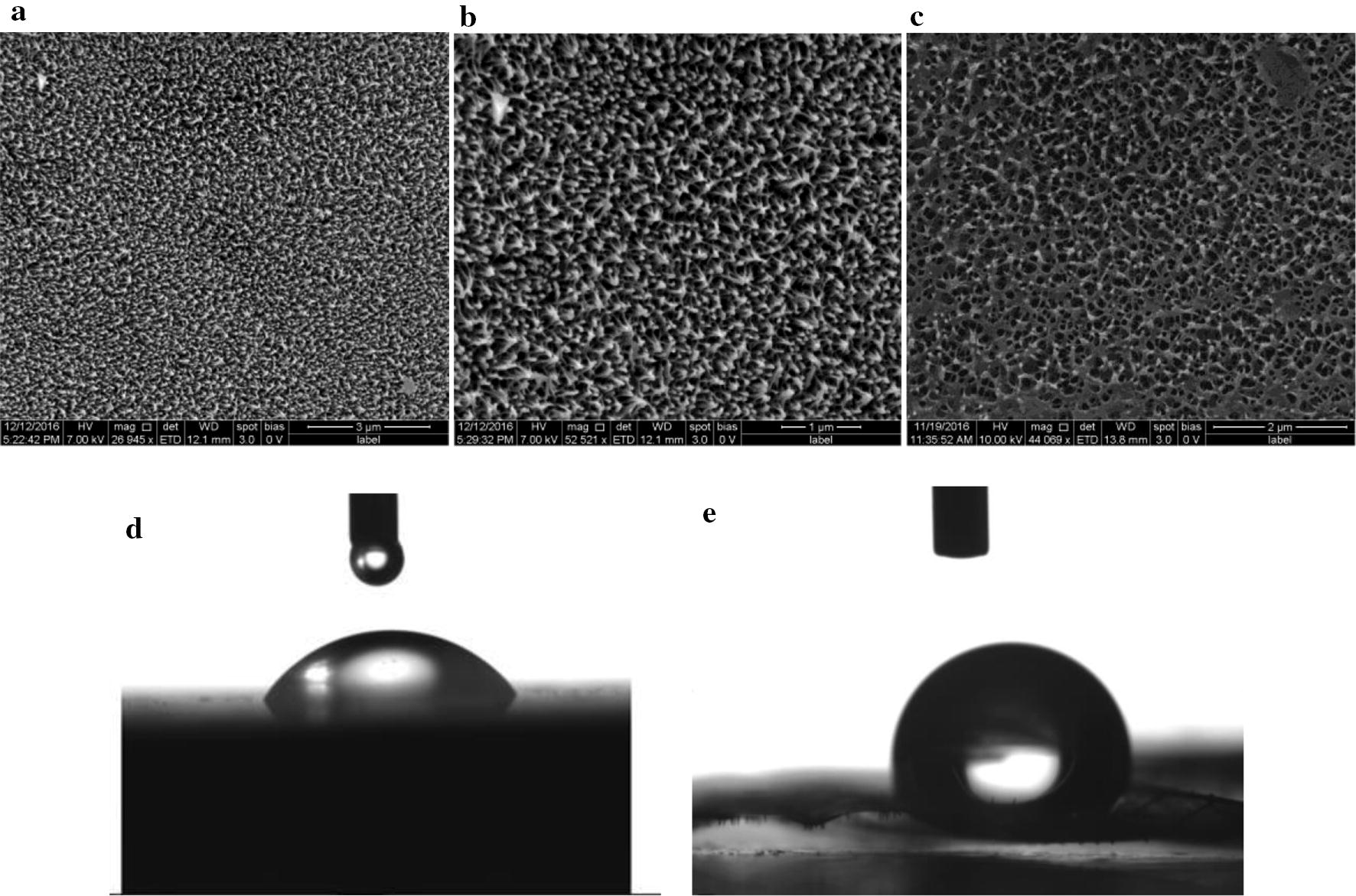
**a** FESEM images of the dragonfly wings showing the nanopillar architecture. **b** Tilted view showing the peaks that were approximately 188 nm tall and, clustering of the peak tips was observed. **c** Top view—distance between the peaks ranged from 88 nm to 157 nm, due to clustering of peak tips. Water Contact angle of the glass cover slip 57.3° (**d**) and *P. flavescens* wing 124° (**e**) obtained using KRUSS drop shape Analyzer DSA

Photographic image of the dragonfly (*Pantala flavescens*) is shown in Additional file 1: Figure S1. The surface topography of the wing was characterized using FESEM and water contact angle measurement (Fig. 1). The FESEM images of the wing surface showed the presence of 120 ± 8 nanopillars per µm^2^ area. Nanopillars were 188 nm tall and spaced 88–157 nm apart due to clustering of peak tips. Wing had the water contact angle of 124°. Presence of long chain fatty acids (Ivanova et al. 2013) and chitin (Rahman and Harfar 2014) were confirmed using Energy Dispersive X-ray spectroscopy (EDAX) and Fourier Transform Infrared Spectroscopy (FTIR) (Additional file 1: Figures S2, S3).

**Fig. 2:**
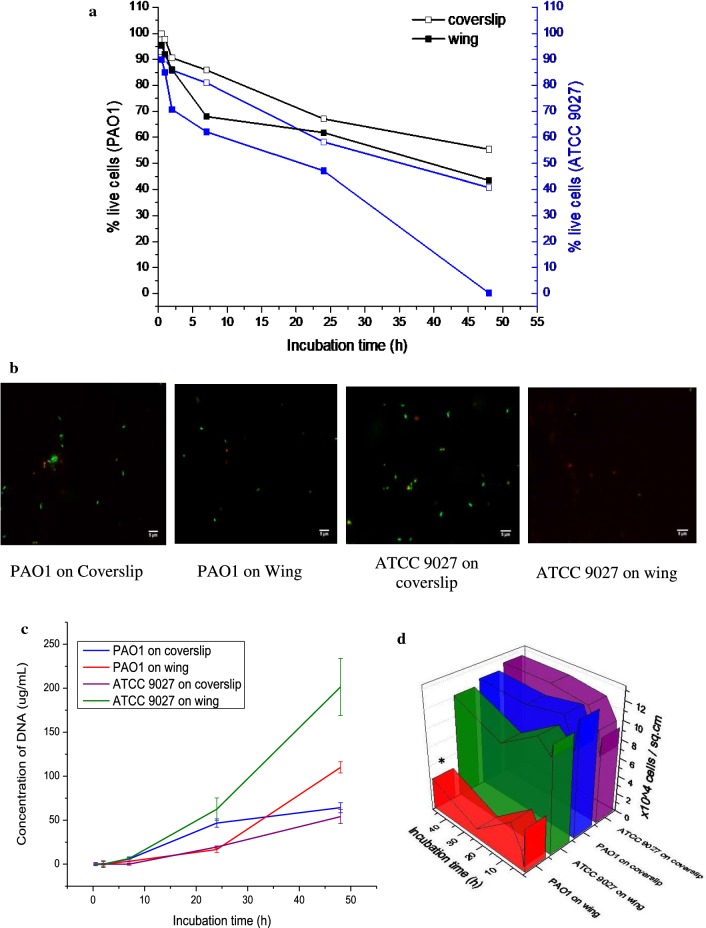
**a** Quantitative viability analyses of *P. aeruginosa* ATCC 9027 and PAO1 on coverslip and wing using flow cytometer. **b** Fluorescent microscopic images of *P. aeruginosa* stained with SYTO9 and propidium iodide. Live cells appear green and, dead cells appear red. **c** Quantity of DNA in the *P. aeruginosa* suspension incubated with glass coverslip and dragonfly wings for discrete time intervals. **d** Number of *P. aeruginosa* cells attached to coverslip and wings enumerated using hemocytometer after incubation for 30 min through 48 h. *Represents statistical significance p < 0.05 using paired t-test

Quantitative analysis of cell viability using flow cytometry (Fig. 2a) showed that 95.54% PAO1 and 89.93% ATCC 9027 were viable 30 min post incubation with wing. The number of live cells declined to 43.47% PAO1 and 0.2% ATCC 9027 at the end of 48 h incubation with wing. Meanwhile, on the coverslip, 99.81% PAO1 and 92.99% ATCC 9027 cells were viable 30 min post incubation, which reduced to 55.43% PAO1 and 40.76% ATCC 9027 at the end of 48 h.

Bacterial viability on the wings of dragonfly was qualitatively analyzed by fluorescent staining. *P. aeruginosa* on wings and coverslip were stained with BacLight live/dead viability kit and imaged using fluorescent microscope (Fig. 2b). Nucleic acid of the cells with intact cell walls take up SYTO 9 and appear green, while the nucleic acid of cells with compromised cell wall integrity appear red by taking up propidium iodide stain. Number of dead cells was higher in ATCC 9027 than PAO1, when incubated with wing.

DNA released due to cell wall damage in the suspension is considered as an indirect measure of dead cells. The DNA explicitly in the suspension was precipitated and quantified, without damaging the live cells with intact cell walls. After 48 h incubation, the amount of DNA released by PAO1 on coverslip and wings were 44.04 ± 5.7 µg/mL and 109.99 ± 6.33 µg/mL respectively. On the other hand, ATCC 9027 released 64.17 ± 8.03 µg/mL and 201.42 ± 32.53 µg/mL of DNA respectively when incubated with coverslip and wing. Increased concentration of DNA was observed in the cell suspensions on the wings than on the coverslip in both the strains (Fig. 2c).

Enumeration of *P. aeruginosa* attached on coverslip/wing (Fig. 2d) indicated that 11.7 ± 2.040 × 10^4^ PAO1 and 12.42 ± 1.873 × 10^4^ ATCC 9027 were attached to 1 × 1 cm^2^ of the coverslip, while the number of cells attached to 1 × 1 cm^2^ wings were 3.8 ± 1.363 × 10^4^ PAO1 and 11.11 ± 2.218 × 10^4^ ATCC 9027. The result indicates that the number of PAO1 attached to wing were three times lesser than the ATCC 9027.

**Fig. 3:**
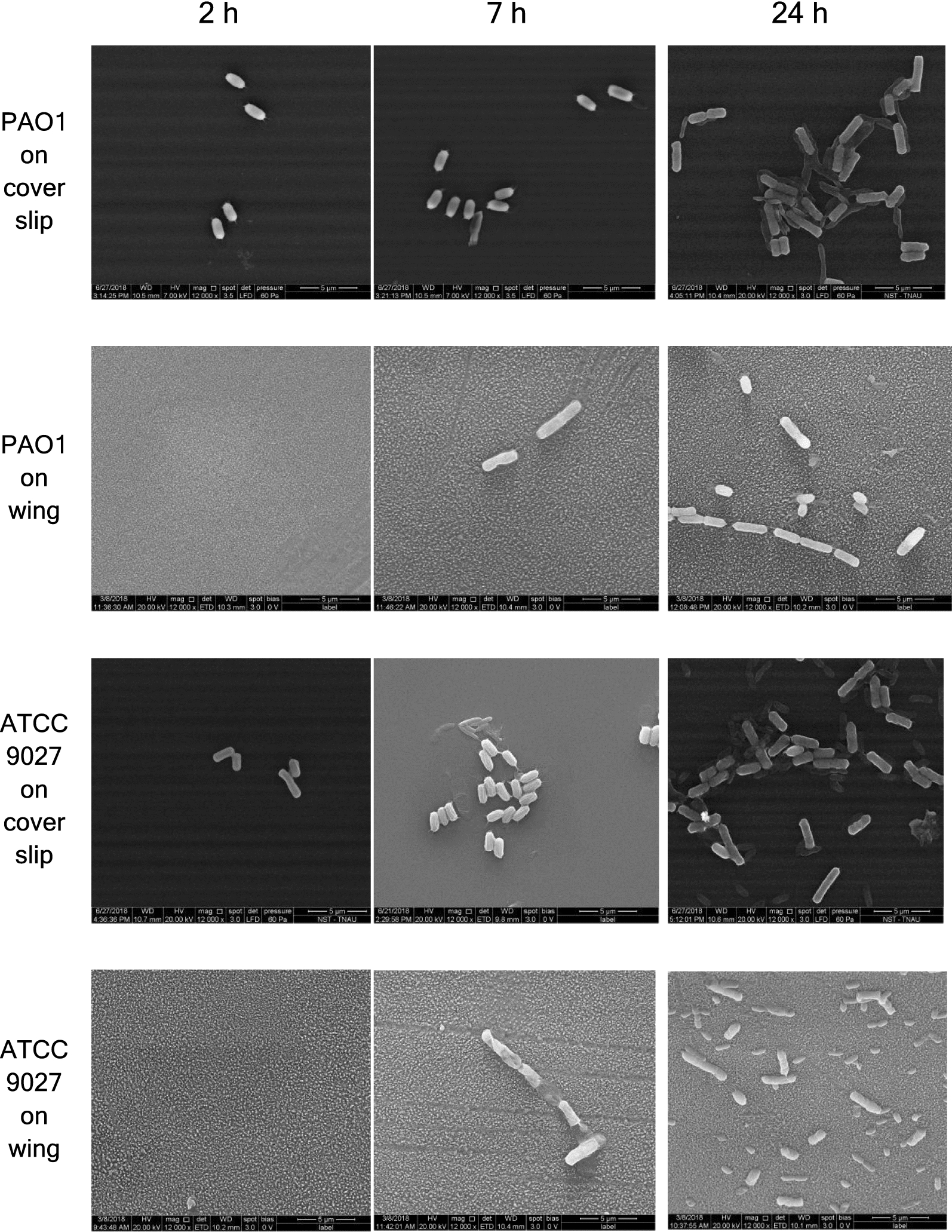
FESEM images of *P. aeruginosa* ATCC 9027 and PAO1 on glass cover slip and on the wing of *Pantala flavescens*. Magnification—×12,000 and bars represent 5 µm

*Pseudomonas aeruginosa* strains attached to coverslip/wing were visualized and imaged under scanning electron microscope (Fig. 3). *P. aeruginosa* cells were observed to be elongated on the surface of the wing compared to the coverslip. The length of *P. aeruginosa* strains ATCC 9027 and PAO1 on the coverslips ranged from 1.672 to 1.827 µm and 1.762 to 2.157 µm respectively. Their lengths changed to 2.063 to 5.332 µm and 3.593 to 5.624 µm respectively on the wings. The widths of ATCC 9027 and PAO1 on the coverslip were 0.776–0.788 µm and 0.708–0.882 µm respectively. While on the wings, their widths changed to 0.925–1.177 µm and 0.868–1.227 µm respectively.

**Fig. 4:**
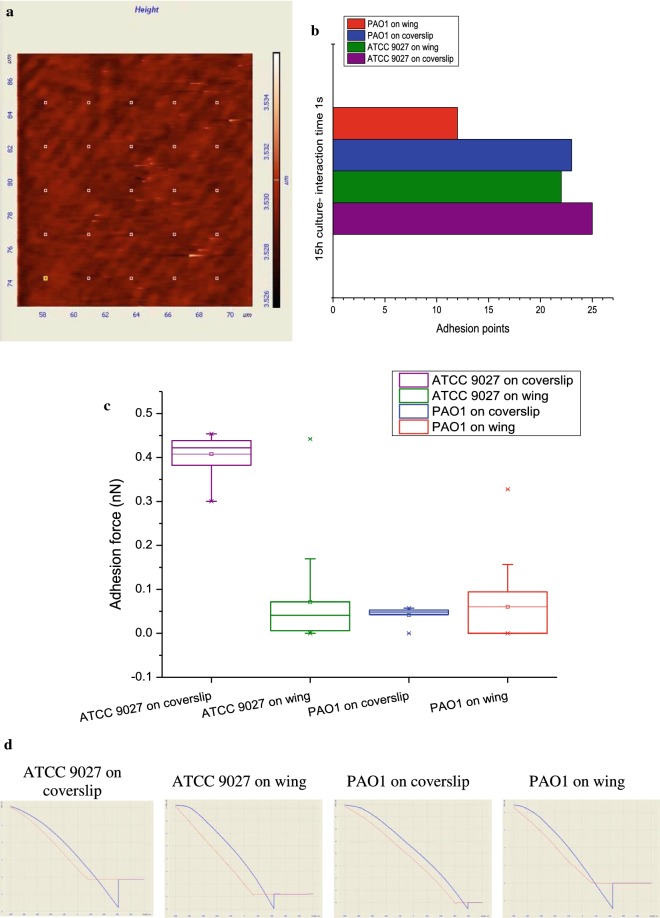
**a** Representative image showing the random 25 regions (white square dots) chosen on the surface of wing to measure the adhesion propensity of the *P. aeruginosa* strains. **b** Number of points on the surface of coverslip and wing to which PAO1 and ATCC 9027 showed adhesion. **c** Adhesion force exerted by ATCC 9027 and PAO1 on coverslip and wing was represented using Box–Whisker plot. Box represents the distribution range of values. Dark line inside the box represents 50% values. Thin line in the box represents average value. Square dot represents median value and, the bars represent standard deviation. **d** Representative force-distance curves of PAO1 and ATCC 9027 on coverslip and wing surface. Red line represents the probe-approach and blue line represents the probe-retraction

Adhesion being the first step towards bacterial attachment (Dunne 2002), the adhesion forces exerted by *P. aeruginosa* strains on glass coverslip (control) and wing surface were measured using Scanning probe microscope. Overnight grown *P. aeruginosa* were attached to the tip of the cantilever and tested for its adhesion on 25 random points (Fig. 4a) chosen on the wing surface and coverslip. The cantilever was allowed to interact with wing/coverslip for 1 s (Touhami et al. 2006). The results (Fig. 4b) indicated that ATCC 9027 showed adhesion to 100% regions on coverslip and 88% regions tested on wing surface, whilst PAO1 showed adhesion to 92% regions on coverslip and 48% regions tested on wing surface. Besides, adhesion force exerted by the *P. aeruginosa* strains on the coverslip and wing were also calculated. The results of adhesion forces (Fig. 4c) indicate that ATCC 9027 exerted a force of 0.4 ± 0.041 nN and 0.08 ± 0.00826 nN on coverslip and wing respectively. PAO1, on the other hand, exerted adhesion forces of 0.05 ± 0.00509 nN and 0.13 ± 0.02277 nN respectively on coverslip and wing. Representative force-distance curves were presented in Fig. 4d.

**Fig. 5:**
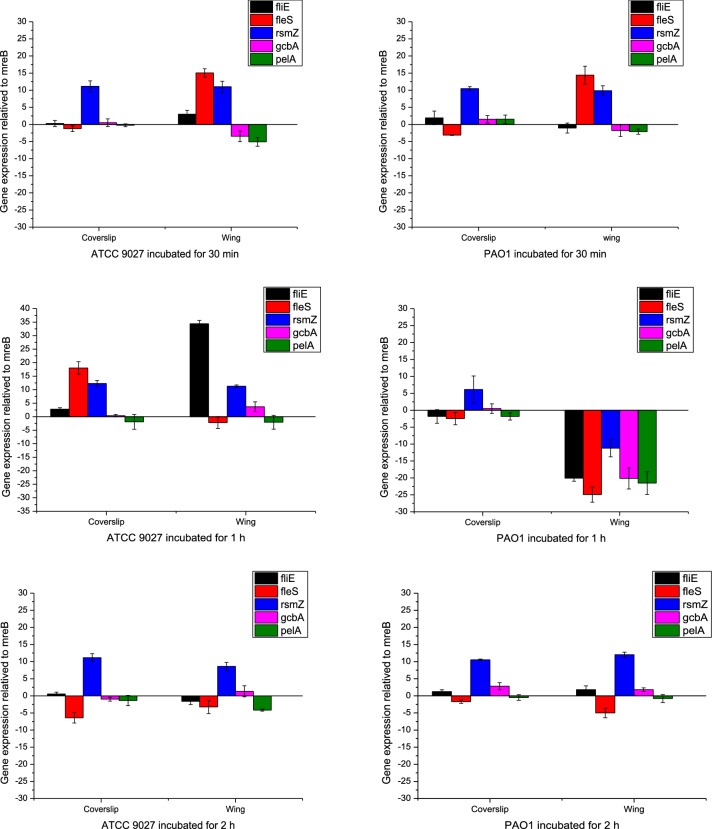
Transcriptional profile of *P. aeruginosa* genes that aid in motility (fliE, fleS), reversible to irreversible attachment (gcbA, rsmZ) and EPS synthesis gene (pelA) carried out using RT-PCR. The expressions were relatived to the constitutive actin-homolog (mreB)

To further investigate the attachment at the transcriptional level, quantitative real-time PCR was carried out for set of 6 genes including the constitutive gene, mreB (actin-homologue). Genes that play a role in flagellar motility (fliE and fleS), Extracellular Polysaccharide (EPS) production (pelA), initial adhesion and transition from reversible to irreversible attachment (gcbA and rsmZ) were analyzed (Fig. 5).

PAO1 when incubated with coverslip, expressed fliE (structural protein in flagellar basal-hook) at lower level, which could be due to the minimal bacterial multiplication as bacteria were transferred from nutrient broth to PBS. However, the expression of fleS (signal transduction in flagella) is reduced, indicating restricted motility. Expression of gcbA that is responsible for reversible to irreversible attachment of bacteria was observed to increase with increase in the incubation time. Expression of rsmZ, a non-coding RNA regulator that assists gcbA in facilitating bacterial attachment was expressed 12 times higher than mreB at 2 h. Upregulation of gcbA and rsmZ indicated that PAO1 likely initiated its attachment to glass coverslip.

PAO1 when incubated with wing, showed reduced expression of fliE till 1 h. Expression of fleS was higher till 30 min, and showed reduction from 1 h. Expression of gcbA was downregulated till 1 h, after which the expression increased. Similar pattern of expression was observed in rsmZ. EPS synthesis gene pelA was much downregulated, which could be due to relatively fewer cells attached on wing surface. PAO1 was observed to have sensed and resisted attachment till 1 h, after which the lower expression of flagellar genes and higher expression of gcbA and rsmZ suggests that PAO1 would have initiated attachment to wing surface after 1 h.

ATCC 9027 post-interaction with coverslip showed slight upregulation in fliE, while the signaling flagellar gene, fleS was downregulated indicating that ATCC 9027 likely initiated attachment to coverslip from 30 min onwards. Expression of gcbA was upregulated, however in lower levels. Expression of rsmZ was 12 to 14 times higher than mreB, insisting that ATCC 9027 showed higher attachment to coverslip than PAO1.

Post-interaction of ATCC 9027 with wing surface, fliE that showed higher expression till 1 h, displayed reduced expression after 1 h. Similarly, the expression of fleS showed drastic reduction for 30 min to 2 h incubation, indicating that motility of ATCC 9027 was greatly reduced, a sign of bacterial attachment. gcbA that showed downregulation at 30 min was upregulated after 30 min. rsmZ also showed 10 times higher expression than mreB, from 30 min strongly insisting that ATCC 9027 was attached to wing surface than PAO1.

## Discussion

Nanopillars on the wings of *Pantala flavescens* showed clustering at the peak tips. Similar observation was reported on the wings of *Diplacodes bipunctata* (Ivanova et al. 2013). Water contact angle measurement suggests that the surface of the dragonfly wing is hydrophobic.

Viability analysis (Fig. 2a, b) of PAO1 and ATCC 9027 suggested that the viability of PAO1 was higher than ATCC 9027 till 48 h on the surface of dragonfly wing. Bacterial cells whose cell walls are damaged on the nanopillars would release the cellular contents into the cell suspension including DNA. It is envisaged that, higher the concentration of DNA in the suspension, higher is the cell death. The DNA quantification data indicated that PAO1 is killed in lesser numbers than ATCC 9027 on wings (Fig. 2c). Cells release DNA under starvation due to autolysis. The DNA quantified in the coverslip (control) could be the result of autolysis. Hence, we presume that the extracellular DNA (eDNA) released due to starvation would be similar on both coverslip and wing. The excess DNA released when the bacteria were incubated with the wing was claimed to be released from the cells that were killed on the wing surface.

Nanopillars could puncture only those cells that are attached to it or in close contact. The viability data cued if the PAO1 restrained itself from attaching to the wing. Enumeration of *P. aeruginosa* attached to coverslip/wing (Fig. 2d) suggested that the number of PAO1 attached to wing were three times lesser than the ATCC 9027, albeit both the *P. aeruginosa* strains PAO1 and ATCC 9027 preferred attaching to coverslip than to wings. Bacterial attachment on the coverslip and wing was imaged using FESEM. Attachment of ATCC 9027 and PAO1 on coverslip were higher than on wing, suggesting that the nanopillar architecture was not conducive for the bacterial adherence. Size of the bacteria was found to increase when they attach to wings than onto the coverslip. This study proves the hypothesis of Pogodin et al. (2013) simulation studies. Unlike the cells on the coverslip, those cells attached to the wings were elongated two folds in the long axis (Fig. 3), which could likely have fetched more focal adhesion points for their attachment. Besides elongation, the width of *P. aeruginosa* ATCC 9027 and PAO1 were found to increase when the cells were attached to wings than on coverslip. The relatively lesser attachment of PAO1 to wings than ATCC 9027 suggested that PAO1 could have possibly restrained itself from attaching to the substrate.

Further adhesion studies were carried out using scanning probe microscopy. From Fig. 4b, it could be observed that PAO1 showed adhesion only to nearly 50% regions on the wing surface, while ATCC 9027 showed adhesion to 90% regions on the wing. PAO1 showed least attachment to the wing surface Though PAO1 showed adhesion to lesser number of regions on the wing surface, the force exerted was relatively higher than on the coverslip. To the coverslip, PAO1 showed adhesion to higher number of regions, but with lesser adhesion force (Fig. 4c, d).

**Fig. 6:**
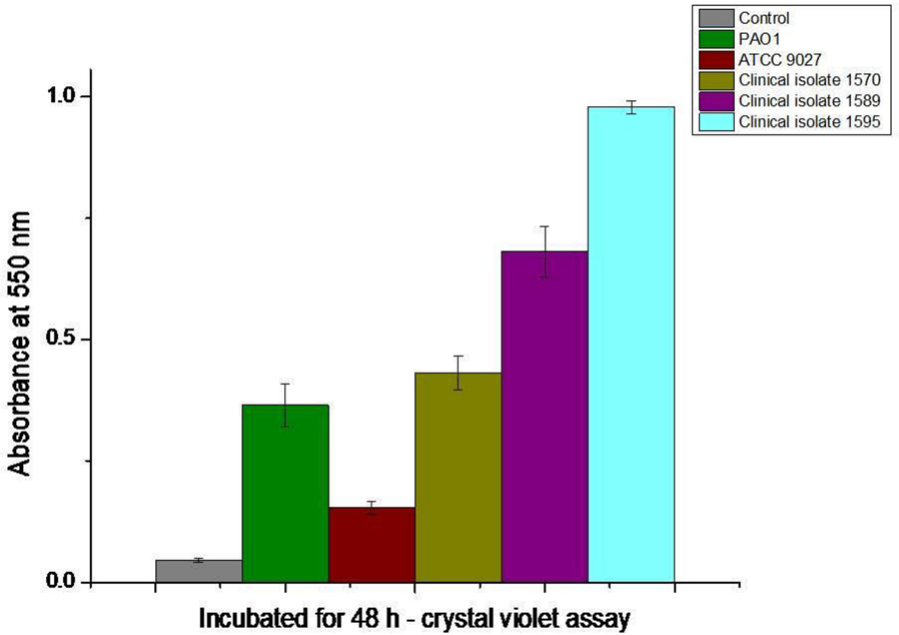
Biofilm forming assay for *P. aeruginosa* strains in nutrient broth for 48 h. Control is un-inoculated nutrient broth

PAO1 being a potent biofilm former (Fig. 6) and virulent strain (Attila et al. 2008), showed lesser adhesion than ATCC 9027. To further explore the possibility of the hypothesis, three *P. aeruginosa* clinical isolates from patients with respiratory ailment were tested for adhesion. All three clinical isolates were potent biofilm formers (Fig. 6) and showed lesser adhesion on the wings of dragonfly (Additional file 1: Figure S4). Based on our studies with PAO1 and three clinical isolates, we propose that virulent strains might restrain from attaching to the wing surface.

*Pseudomonas aeruginosa* ATCC 9027, being an avirulent (Jayal et al. 2017; Grosso-Becerra et al. 2016) and moderate biofilm forming strain (Fig. 6) was killed on the nanopillars of dragonfly wings (Ivanova et al. 2013), wings of Clanger cicada (Ivanova et al. 2012), black silicon (Ivanova et al. 2013) and TiO_2_ surface (Diu et al. 2014; Bhadra et al. 2015). Besides ATCC 9027, *P. aeruginosa* ATCC 9721 (Truong et al. 2017) and ATCC 27853 (Diu et al. 2014; Tripathy et al. 2017) were also killed on the nanopillar topography. On the contrary PAO1 and three clinical isolates showed lesser adhesion propensity to the wing surface (Fig. 4 and Additional file 1: Figure S4) and showed higher survival on the nanopillar topography (Fig. 2a and Additional file 1: Figure S5) of dragonfly wing.

The relatively lesser attachment of PAO1 was analyzed at the transcriptional level using real-time PCR. Generally, the events that occur during bacterial attachment include reduction in motility (fliE, fleS), production of extracellular polysaccharide (pelA) and, expression of genes that aid in attachment and facilitate transition from reversible to irreversible attachment (GcA, rsmZ) (Petrova et al. 2014). Increase in gcbA and rsmZ facilitates bacterial attachment and, reduction in expression of motility genes (fliE and fleS) is a sign of bacteria initiating attachment. Results in Fig. 5, showed that expression of genes aiding attachment was delayed in PAO1 compared to ATCC 9027, suggesting that ATCC 9027 preferred attachment on wing surface than PAO1.

In conclusion, *P. aeruginosa* PAO1 and tested clinical isolates showed higher survival and lesser adhesion to the wing surface unlike ATCC 9027. Based on the viability and attachment results, the study suggests that virulent strains of *P. aeruginosa* may evade attachment on wing surface. Transcriptional level analyses were found supportive to the above results. The study demonstrated the bacterial strategy to overcome the bactericidal effect of dragonfly wing surface. This finding has not been reported so far in the scientific literatures, which makes it novel. With the progress in the development of implants with bioinspired surfaces, the findings of this study will make the scientific community to relook the strategy of preventing bacterial associated infections in implants.

## Supplementary information

**Additional file 1.** Additional figures.

## Data Availability

All the experimental protocols were from published sources and could be replicated. The clinical strains used in the current study are not publicly available. Strains shall be available upon request and with permission from PSG Institute of Medical Sciences & Research, Coimbatore, India.

## Abbreviations

PBS: Phosphate buffered saline
FESEM: Field emission scanning electron microscope
EDAX: Energy dispersive X-ray spectroscopy
FTIR: Fourier transform infra red radiation

## References

[CR1] Asensio G, Vazquez-Lasa B, Rojo L (2019). Achievements in the topographic design of commercial titanium dental implants: towards anti-peri-implantitis surfaces. J Clin Med.

[CR2] Asker D, Awad TS, Baker P, Howell PL, Hatton BD (2018). Non-eluting, surface-bound enzymes disrupt surface attachment of bacteria by continuous biofilm polysaccharide degradation. Biomaterials.

[CR3] Attila C, Ueda A, Cirillo SLG, Cirillo JD, Chen W, Wood TK (2008). *Pseudomonas aeruginosa* PAO1 virulence factors and poplar tree response in the rhizosphere. Microb Biotechnol.

[CR4] Bandara CD, Singh S, Afara IO, Wolff A, Tesfamichael T, Ostrikov K, Oloyede A (2017). Bactericidal effects of natural nanotopography of dragonfly wing on *Escherichia coli*. ACS Appl Mater Interface.

[CR5] Bhadra CM, Truong VK, Pham VTH, Al Kobaisi M, Seniutinas G, Wang JY, Juodkazis S, Crawford RJ, Ivanova EP (2015). Antibacterial titanium nano-patterned arrays inspired by dragonfly wings. Sci Rep.

[CR6] Chough H, Calvin L, Cesar DR, Brendon BT, Mai TY, Yee AF, Cai J (2018) Fabrication of nanopatterns on an artificial cornea device

[CR7] Dickson MN, Liang EI, Rodriguez LA, Vollereaux N, Yee AF (2015). Nanopatterned polymer surfaces with bactericidal properties nanopatterned polymer surfaces with bactericidal properties. Biointerphases.

[CR8] Diu T, Faruqui N, Sjöström T, Lamarre B, Jenkinson HF, Su B, Ryadnov MG (2014). Cicada-inspired cell-instructive nanopatterned arrays. Sci Rep.

[CR9] Dunne WM (2002). Bacterial adhesion: seen any good biofilms lately?. Clin Microbiol Rev.

[CR10] Fisher LE, Yang Y, Yuen M-F, Zhang W, Nobbs AH, Su B (2016). Bactericidal activity of biomimetic diamond nanocone surfaces. Biointerphases.

[CR11] Grosso-Becerra MV, González-Valdez A, Granados-Martínez MJ, Morales E, Servín-González L, Méndez JL, Delgado G, Morales-Espinosa R, Ponce-Soto GY, Cocotl-Yañez M, Soberón-Chávez G (2016). *Pseudomonas aeruginosa* ATCC 9027 is a non-virulent strain suitable for mono-rhamnolipids production. Appl Microbiol Biotechnol.

[CR12] Hasan J, Webb HK, Truong VK, Pogodin S, Baulin VA, Watson GS, Watson JA, Crawford RJ, Ivanova EP (2013). Selective bactericidal activity of nanopatterned superhydrophobic cicada *Psaltoda claripennis* wing surfaces. Appl Microbiol Biotechnol.

[CR13] Hassan A, Usman J, Kaleem F, Omair M, Khalid A, Iqbal M (2011). Evaluation of different detection methods of biofilm formation in the clinical isolates. Braz J Infect Dis.

[CR14] Hayes MJ, Levine TP, Wilson RH (2016). Identification of Nanopillars on the Cuticle of the Aquatic Larvae of the Drone Fly (Diptera: Syrphidae). J Insect Sci.

[CR15] Herzenberg LA, Tung J, Moore WA, Herzenberg LA, Parks DR (2006). Interpreting flow cytometry data: a guide for the perplexed. Nat Immunol.

[CR16] Ivanova EP, Hasan J, Webb HK, Truong VK, Watson GS, Watson JA, Baulin VA, Pogodin S, Wang JY, Tobin MJ, Löbbe C, Crawford RJ (2012). Natural bactericidal surfaces: mechanical rupture of *Pseudomonas aeruginosa* cells by cicada wings. Small.

[CR17] Ivanova EP, Hasan J, Webb HK, Gervinskas G, Juodkazis S, Truong VK, Wu AHF, Lamb RN, Baulin VA, Watson GS, Watson JA, Mainwaring DE, Crawford RJ (2013). Bactericidal activity of black silicon. Nat Commun.

[CR18] Jayal A, Johns BE, Purdy KJ, Maddocks E (2017). Draft genome sequence of *Pseudomonas aeruginosa* ATCC 9027, originally isolated from an outer ear infection. Genom Announ.

[CR19] O’Toole GA (2011). Microtiter dish biofilm formation assay. J Vis Exp.

[CR20] Orapiriyakul W, Young PS, Damiati L, Tsimbouri PM (2018). Antibacterial surface modification of titanium implants in orthopedics. J Tissue Eng.

[CR21] Petrova OE, Kathryn EC, Karin S (2014). The *P. aeruginosa* diguanylate cyclase GcbA, a homolog of the *P. fluorescens* GcbA, promotes initial attachment to surfaces, but not biofilm formation, via regulation of motility. J Bacteriol.

[CR22] Pogodin S, Hasan J, Baulin VA, Webb HK, Truong VK, Phong Nguyen TH, Boshkovikj V, Fluke CJ, Watson GS, Watson JA, Crawford RJ, Ivanova EP (2013). Biophysical model of bacterial cell interactions with nanopatterned cicada wing surfaces. Biophys J.

[CR23] Prabu P, Kim KW (2008). Antimicrobial drug release scaffolds of natural and synthetic biodegradable polymers. Macromol Res.

[CR24] Rahman MA, Halfar J (2014) Coralline algae : new insights into the compactum. 10.1038/srep06162 10.1038/srep06162 PMC4141250 25145331

[CR25] Release N (2018) WHO publishes list of bacteria for which new antibiotics are urgently needed WHO priority pathogens list for R & D of new antibiotics, pp 1–7

[CR26] Romanò CL, Sara S, Enrico G, Delia R, Lorenzo D (2015). Antibacterial coating of implants in orthopaedics and trauma: a classification proposal in an evolving panorama. J Orthop Surg Res.

[CR27] Swartjes JJTM, Das T, Sharifi S, Subbiahdoss G, Sharma PK, Krom BP, Busscher HJ, Van Der Mei HC (2013). A functional dnase i coating to prevent adhesion of bacteria and the formation of biofilm. Adv Funct Mater.

[CR28] Tang S, Tan H, Lin W, Wang Y (2014). Preparation, characterization, and in vitro osteoblast functions of a nano-hydroxyapatite/polyetheretherketone biocomposite as orthopedic implant material. Int J Nanomedicine.

[CR29] Tiller JC, Liao CJ, Lewis K, Klibanov AM (2001). Designing surfaces that kill bacteria on contact. PNAS.

[CR30] Touhami A, Jericho MH, Boyd JM, Beveridge TJ (2006). Nanoscale characterization and determination of adhesion forces of *Pseudomonas aeruginosa* pili by using atomic force microscopy. J Bacteriol.

[CR31] Tripathy A, Sen P, Su B, Briscoe WH (2017). Natural and bioinspired nanostructured bactericidal surfaces. J Colloid Interface Sci.

[CR32] Truong VK, Geeganagamage NM, Baulin VA, Vongsvivut J, Tobin MJ, Luque P, Crawford RJ, Ivanova EP (2017). The susceptibility of *Staphylococcus aureus* CIP 65.8 and *Pseudomonas aeruginosa* ATCC 9721 cells to the bactericidal action of nanostructured *Calopteryx haemorrhoidalis* damselfly wing surfaces. Appl Microbiol Biotechnol.

[CR33] Yi G, Yuan Y, Li X, Zhang Y (2018). ZnO nanopillar coated surfaces with substrate-dependent superbactericidal property. Small.

