# Supplementary material for: Survival strategy of Pseudomonas aeruginosa on the nanopillar topography of dragonfly (Pantala flavescens) wing
Source: AMB Express. 2020 May 6;10:85. doi: 10.1186/s13568-020-01021-7 (PMC7203277; doi:10.1186/s13568-020-01021-7)
Supplement: Supplementary file 1 — Additional file 1. Additional figures. [file 13568_2020_1021_MOESM1_ESM.docx]

Additional file 1

**AMB Express**

**Survival strategy of Pseudomonas aeruginosa on the nanopillar topography of dragonfly (Pantala flavescens) wing**

Banu Pradheepa Kamarajan and Ananthasubramanian Muthusamy *

Department of Biotechnology, PSG College of Technology, Coimbatore, TamilNadu, India

*corresponding author

email – [biosubramanian@gmail.com](mailto:biosubramanian@gmail.com)

Telephone – 0422 257 2177

Fax - 0422 2573833


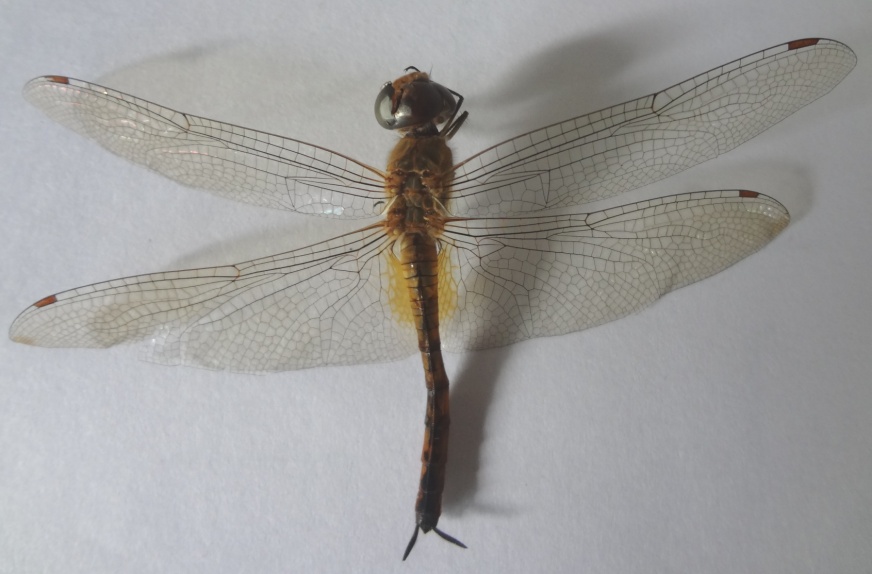


Figure S1. Photographic image of *P. flavescens*, captured using SONY CYBER-SHOT (Model no. DSC-WX50) 16.2 MP camera, showing the rusty thorax, segmented abdomen, transparent wings with stigma on the all the four wings and, hind wing with characteristic yellow color.


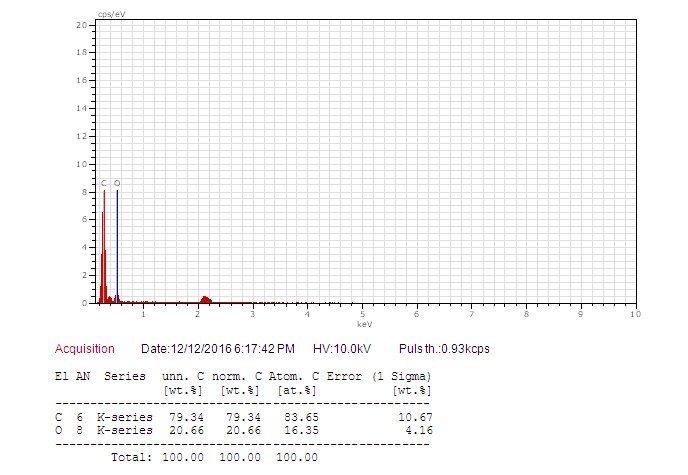


Figure S2. EDAX of *P. flavescens* wing showing 83.65 (at %) of C and 16.35 (at %) of O.

**
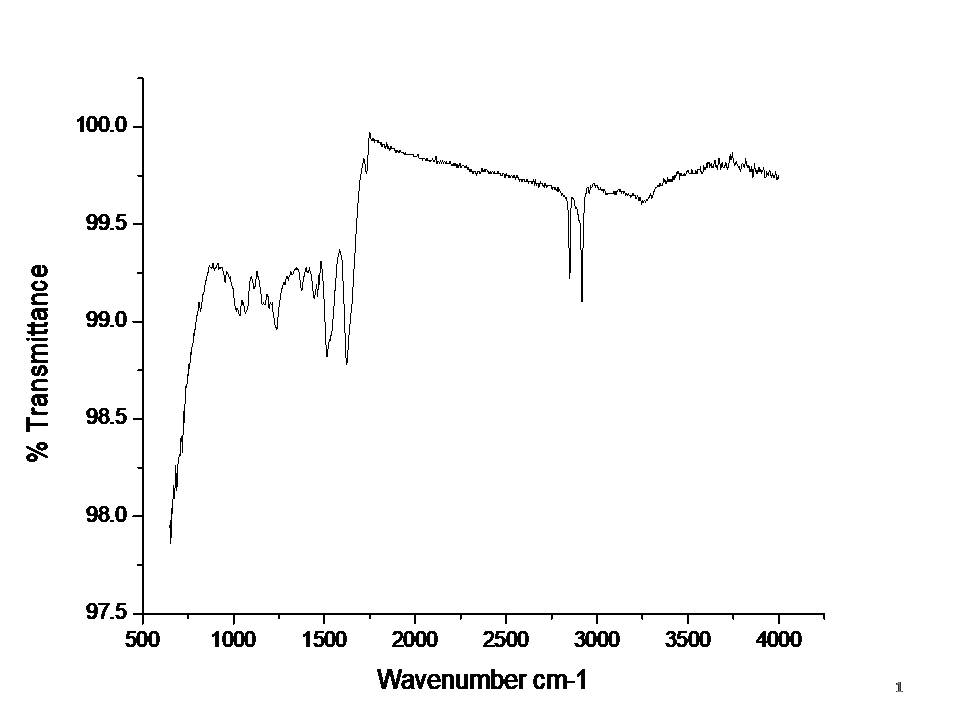
**

Figure S3. FTIR spectra of dragonfly wing

b

a

Figure S4. (a) Number of points to which 15 h cultures of *P. aeruginosa* clinical isolates show adhesion and (b) their respective mean adhesion forces, when allowed to interact with coverslip/wing for 1 second.


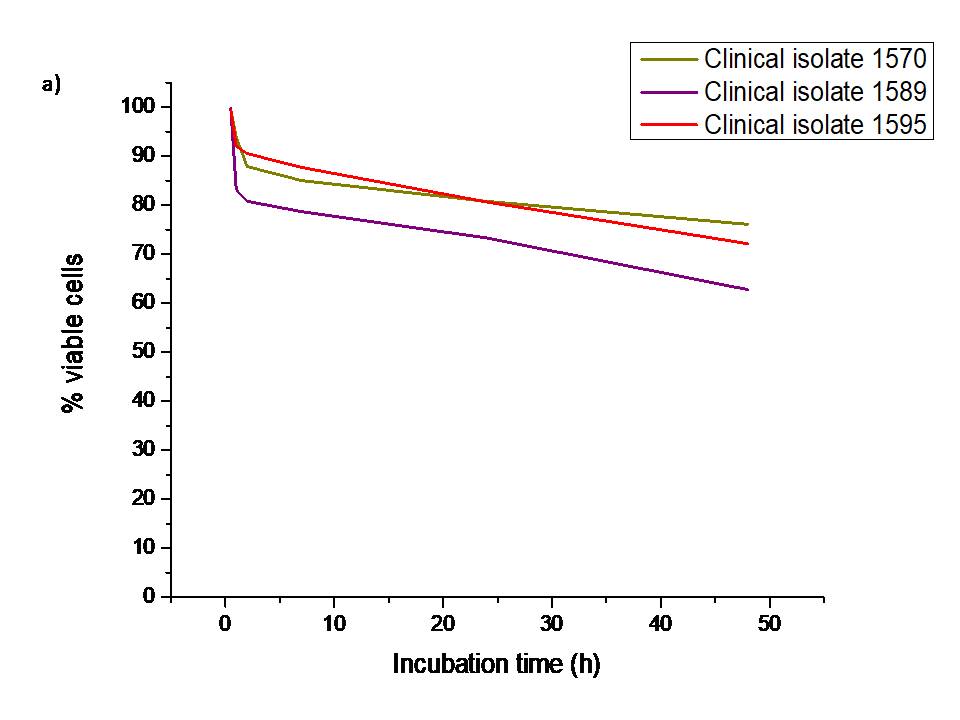

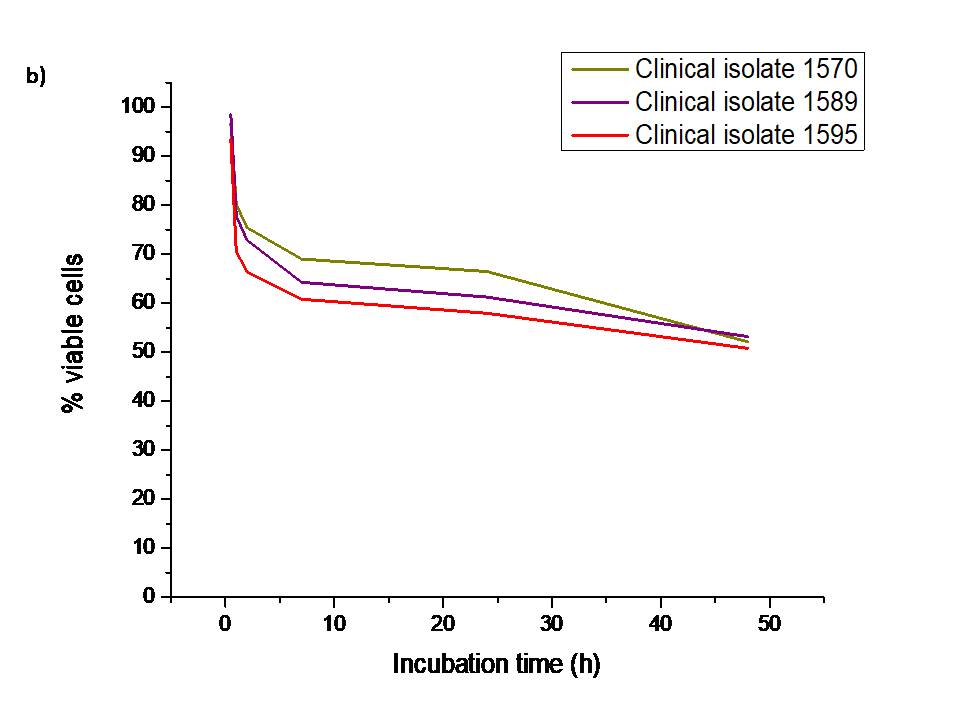


Figure S5. Viability of clinical isolates on the coverslip (a) and wing (b) analyzed using flow cytometer.
